# Accelerometer-Measured Physical Activity Changes After Transitions to Concern About Falling, a Recent Fall, or Both in Older Adults: A Longitudinal NHATS Study

**DOI:** 10.21203/rs.3.rs-10692562/v1

**Published:** 2026-09-01

**Authors:** Dejin Wang, Jueliang Tian, Yuxi Peng, Shaoxue Wu, Yuhuan Feng

**Affiliations:** School of Medicine and Science, Zunyi Medical University; State University of Jakarta; State University of Jakarta; State University of Jakarta; Ningxia Normal University

**Keywords:** accelerometry, concern about falling, falls, physical activity, activity fragmentation, older adults

## Abstract

**Background:**

Whether recent falls and concern about falling represent distinct precursors of subsequent free-living activity change remains unclear.

**Methods:**

We analyzed National Health and Aging Trends Study Rounds 11–14 using a person-interval design spanning three annual intervals, with each interval restricted to participants who reported neither a recent fall nor concern about falling at the preceding wave. Follow-up states were classified as stable neither, concern only, fall only, or both. Wrist accelerometry provided measures of activity volume and fragmentation, with active minutes per day as a secondary outcome. Survey-weighted models adjusted for the preceding outcome, demographic and health covariates, prior-year falls, and interval; Holm correction was applied across the two primary global tests.

**Results:**

The analytic sample included 873 person-intervals from 437 participants. Transition state was associated with activity volume (Holm-adjusted P = 0.021), whereas evidence for differences in fragmentation was inconclusive (Holm-adjusted P = 0.080). Relative to stable neither, activity volume was 3.18% lower for concern only (95% CI, −7.47% to 1.32%), 6.95% lower for fall only (95% CI, −14.16% to 0.86%), and 12.41% lower for co-occurring fall and concern (95% CI, −20.80% to −3.14%). The co-occurring state was also associated with 41.13 fewer active minutes/day (95% CI, −71.46 to −10.79). Concern-only and fall-only transitions were not clearly different, and concern-related activity restriction was not clearly associated with larger activity changes.

**Conclusions:**

Co-occurring fall and concern showed the clearest association with subsequent loss of device-measured activity volume, while fragmentation changes remained uncertain.

## Background

Falls in later life are a major source of injury-related morbidity and mortality [1, 2]. Their relevance extends beyond the event itself because changes in mobility and confidence can alter how older adults move through daily life. Regular physical activity remains central to healthy aging, and World Health Organization guidance recommends that older adults, including those with chronic conditions or disability, remain as active as their abilities allow [3]. A fall-related change may therefore be clinically important even without major injury if it is followed by a reduction in everyday movement.

Recent falls and concern about falling are related but not interchangeable. In longitudinal NHATS data, concern about falling predicted subsequent limitations in mobility, self-care, and household activities after accounting for previous falls, while multiple previous falls retained independent associations after accounting for concern [4]. Concern also does not uniformly translate into activity restriction. In a prospective cohort, new concern-related restriction developed in only a subset of older adults and was more likely among those with greater frailty, poorer functional mobility, or an intervening fall [5]. These distinctions support examining concern-only, fall-only, and co-occurring states separately rather than treating them as a single fall-related exposure.

Objective monitoring has linked concern about falling to lower physical activity in older adults, whereas associations with falls have been less consistent and longitudinal evidence remains limited [6, 7]. Among older adults with glaucoma, injurious falls were associated with steeper declines in accelerometer-measured steps and active minutes over follow-up [8]. In a separate analysis, multiple falls were associated with declining active minutes and greater activity fragmentation [9]. In NHATS, greater activity accumulation and lower fragmentation at Round 11 were associated with lower subsequent fall risk at Round 12, providing longitudinal evidence in the opposite temporal direction [10]. NHATS research has also examined four combinations of falls and fear of falling in relation to later functional limitations [11]. What remains unresolved is whether, among older adults recently free of both falls and concern, transitions to concern only, a fall only, or both are associated with distinct subsequent changes in device-measured free-living activity compared with remaining free of both.

Using repeated NHATS data from Rounds 11–14, we examined person-interval transitions from a prior-wave state of neither recent fall nor concern about falling to stable neither, concern only, fall only, or both. The primary outcomes were subsequent changes in total activity volume and activity fragmentation measured by wrist accelerometry; active minutes per day was examined as a secondary outcome. We also assessed whether concern-related activity restriction distinguished a subgroup with greater subsequent activity change within concern-only transitions. Because the data are observational and transition states are ascertained at annual interviews, the analysis estimates adjusted longitudinal associations rather than causal or precise-onset effects.

## Methods

### Study design and analytic sample

The National Health and Aging Trends Study (NHATS) is an ongoing longitudinal study based on a national probability sample of U.S. Medicare beneficiaries aged 65 years and older [12]. We used public-use data from Rounds 11–14 (2021–2024), when wrist accelerometry was collected repeatedly, and organized observations into person-intervals spanning three consecutive annual intervals: R11–R12, R12–R13, and R13–R14. For each interval, eligibility was restricted to participants who reported neither a fall in the previous month nor concern about falling at the preceding wave. Transition status was then defined at the next interview, before the subsequent accelerometry monitoring period.

An eligible person-interval required ascertainable transition status, at least four valid accelerometer days at both interval endpoints, observed values for the corresponding physical activity measure at both endpoints, complete primary covariates, and valid survey-design variables. Participants could contribute more than one interval when they again met the preceding-wave eligibility criterion. The final primary analytic sample comprised 873 person-intervals from 437 unique participants.

### Measures

Transition states. Recent falls were identified from self-reported falls during the previous month [10], and concern about falling was defined by reported worry about falling during the same period [4]. At each follow-up wave, participants were classified as neither, concern only, fall only, or both. Because every interval began in the neither state, these categories represented stable neither, transition to concern only, transition to fall only, and transition to both; stable neither was the reference group. Fall history during the previous year was retained separately as a baseline covariate. Among concern-only transitions, concern-related activity restriction was defined by reporting that worry about falling limited usual activities.

Physical activity outcomes. NHATS participants wore a triaxial ActiGraph CentrePoint Insight Watch on the nondominant wrist for eight days. Raw acceleration was sampled at 64 Hz and processed by NHATS into one-minute epochs and round-specific summary measures [10]. A valid day required more than 90% daily wear time, and the primary analysis required at least four valid days at both interval endpoints. The two primary outcomes were log total activity counts, representing overall activity volume, and active-to-sedentary transition probability (ASTP), representing activity fragmentation. ASTP is the probability of transitioning from an active state to a sedentary state and is equivalent to the inverse of mean active-bout length; higher values indicate more fragmented activity [13]. Active minutes per day, defined by the NHATS summary metric as minutes above 1,853 counts/min, was a secondary outcome [10].

Covariates. Covariates were measured at the preceding wave of each interval and included age (represented by the six-level NHATS category of 65–69, 70–74, 75–79, 80–84, 85–89, and ≥ 90 years and entered as a single ordinal term), sex, a count of nine chronic conditions, episodic memory, depressive symptoms, physical performance, and prior-year fall history. The multimorbidity count included heart disease, hypertension, arthritis, osteoporosis, diabetes, lung disease, stroke, dementia or Alzheimer disease, and cancer. Episodic memory was the sum of immediate and delayed word recall scores (0–20). Depressive symptoms were measured with the two-item Patient Health Questionnaire, and physical performance was represented by the NHATS Short Physical Performance Battery score. The corresponding baseline physical activity measure and interval indicator were included directly in each outcome model.

### Statistical analysis

All analyses incorporated the NHATS complex sample design. Survey objects were specified using the accelerometry full-sample weight and accelerometry stratum from the endpoint round of each person-interval. Respondent identifier was used as the clustering unit in the primary analysis to account for repeated contributions from the same participant, and the survey design was defined before restriction to the analytic domain. Descriptive estimates were survey weighted.

For each primary outcome, we fitted a survey-weighted Gaussian regression model with the follow-up physical activity measure as the dependent variable. Transition state was the exposure, with stable neither as the reference, and models adjusted for the corresponding baseline outcome, age, sex, multimorbidity, episodic memory, depressive symptoms, physical performance, prior-year fall history, and interval. The overall contribution of transition state was evaluated with a three-degree-of-freedom Wald test. Holm correction was applied across the two primary global tests for activity volume and activity fragmentation. The three contrasts against stable neither were reported with 95% confidence intervals irrespective of the global test result.

Coefficients and confidence limits from models of log total activity counts were transformed as 100[exp(β) − 1] and reported as percentage differences. ASTP was analyzed on its percentage scale, so model coefficients were reported directly as percentage-point differences. Active minutes were reported in minutes per day. All confidence intervals were two-sided at the 95% level. Primary analyses used complete observations for the transition state, paired outcome values, primary covariates, and survey-design variables. Analyses were conducted in R version 4.6.1 using the survey package version 4.5.

### Secondary and sensitivity analyses

Active minutes per day were analyzed as a secondary outcome using the same model, adjustment set, and global transition-state test as the primary outcomes, but were not included in the Holm correction. A prespecified secondary contrast compared concern-only with fall-only transitions; interpretation emphasized the estimate and 95% confidence interval rather than an additional significance threshold. Within concern-only transitions, we also compared participants who did and did not report concern-related activity restriction using the same covariate-adjusted model.

Sensitivity analyses were limited to the two primary outcomes. We repeated the primary models using a minimum of three valid accelerometer days at both endpoints, fitted direct change-score models, and omitted each annual interval in turn. We also replaced respondent-level clustering with the NHATS geographic variance unit. To examine covariate robustness, additional models separately added race/ethnicity, log-transformed household income, and both variables to the primary adjustment set. Race/ethnicity was represented by three categories (non-Hispanic White, non-Hispanic Black, and other/Hispanic/multiracial), and household income was transformed as log(1 + income). Race/ethnicity was reserved for sensitivity analysis because of sparse cells in the fall-only state, while adding income materially reduced the complete-case sample. The study protocol and statistical analysis plan were preregistered on the Open Science Framework; repository details are withheld from the blinded manuscript and provided to the editors.

## Results

### Analytic sample and transition states

Across 10,274 candidate person-intervals in which the preceding-wave state was neither, transition status was ascertainable for 9,092. Paired accelerometry with at least four valid days at both interval endpoints was available for 945 person-intervals. The primary analytic sample comprised 873 person-intervals with complete prespecified covariates, contributed by 437 participants. Among the final intervals, 670 were stable neither, 140 transitioned to concern only, 36 to fall only, and 27 to both a fall and concern; the corresponding survey-weighted proportions were 76.2%, 16.5%, 4.0%, and 3.3%. Survey-weighted descriptive changes in physical activity are summarized in Table 1, with baseline and follow-up estimates presented in Supplementary Table S1 in Additional file 1.

Baseline covariate distributions and missingness in the paired-accelerometry risk set before complete-case restriction are reported in Supplementary Table S6 in Additional file 1.

### Primary physical activity outcomes

The global transition-state test for activity volume yielded P = 0.011 and Holm-adjusted P = 0.021. Compared with stable neither, estimated activity volume was 3.18% lower for concern only (95% CI, −7.47% to 1.32%) and 6.95% lower for fall only (95% CI, −14.16% to 0.86%). For the co-occurring state, activity volume was 12.41% lower (95% CI, −20.80% to −3.14%), the largest adjusted point estimate among the three contrasts (Table 2).

For activity fragmentation, the global transition-state test yielded P = 0.080 and Holm-adjusted P = 0.080. Adjusted differences in active-to-sedentary transition probability were 1.04 percentage points (pp) for concern only (95% CI, −0.21 to 2.29) and 1.49 pp for fall only (95% CI, −0.85 to 3.83). The estimate for the co-occurring state was 2.09 pp (95% CI, −0.27 to 4.45). All three confidence intervals included zero (Table 2).

### Secondary analyses

For active minutes/day, the global transition-state test yielded P = 0.008; this secondary outcome was not included in the multiplicity adjustment. Relative to stable neither, adjusted differences were − 11.58 min/day for concern only (95% CI, −25.43 to 2.26) and − 21.55 min/day for fall only (95% CI, −45.67 to 2.57). The difference for the co-occurring state was − 41.13 min/day (95% CI, −71.46 to −10.79) (Table 2).

The prespecified concern-only versus fall-only contrast was 4.06% for activity volume (95% CI, −5.05% to 14.04%) and − 0.45 pp for activity fragmentation (95% CI, −3.08 to 2.18). The corresponding difference in active minutes was 9.97 min/day (95% CI, −16.94 to 36.87). Each confidence interval spanned zero (Supplementary Table S2 in Additional file 1).

Within concern-only transitions, the activity-restriction stratum contributed 29 person-intervals from 28 participants, whereas the no-restriction stratum contributed 111 person-intervals from 109 participants. Comparing restriction with no restriction, adjusted differences were − 5.98% for activity volume (95% CI, −14.09% to 2.89%) and 1.37 pp for activity fragmentation (95% CI, −0.95 to 3.69). The active-minutes difference was − 16.69 min/day (95% CI, −46.37 to 12.98). Each confidence interval included zero (Supplementary Table S5 in Additional file 1).

### Sensitivity analyses

The activity-volume contrast between the co-occurring and stable-neither states remained negative in every prespecified sensitivity analysis, ranging from − 15.42% to −7.40% (Supplementary Table S3 in Additional file 1). The estimate was − 11.88% with a three-valid-day threshold, −12.38% in the direct change-score model, −12.41% with geographic variance-unit clustering, and − 14.03% with expanded adjustment for race/ethnicity and household income. Leave-one-interval-out estimates also remained negative across the three annual intervals.

For activity fragmentation, the corresponding contrast remained positive across sensitivity analyses, ranging from 0.95 to 2.88 pp. None of the sensitivity-model global transition tests was below 0.05 (Supplementary Table S4 in Additional file 1).

## Discussion

The principal finding was that transition to a state combining a recent fall with concern about falling was associated with the clearest subsequent reduction in free-living activity volume. Active minutes showed a concordant pattern, whereas evidence for a change in activity fragmentation was less conclusive. Concern-only and fall-only transitions were not clearly distinguishable from one another, and concern-related activity restriction did not identify a subgroup with a larger subsequent activity change.

These findings support considering the co-occurring state separately when examining subsequent activity. In a prospective glaucoma cohort, injurious fallers showed steeper declines in accelerometer-measured steps and active minutes than non-fallers, whereas physical activity did not significantly decline among noninjurious fallers [8]. A separate longitudinal analysis found that multiple, but not single, fallers showed greater declines in active minutes and greater fragmentation over follow-up [9]. In NHATS, concurrent falls and fear of falling have also been associated with higher subsequent risks of mobility, self-care, and household limitations than the neither state [11]. The present findings extend this pattern to device-measured free-living activity among older adults who were free of both recent falls and concern at the preceding wave. The co-occurring state should not, however, be interpreted as a synergistic effect.

Estimates for concern-only and fall-only transitions were imprecise. They do not establish that either state is behaviorally negligible, nor do they support ranking fall-only above concern-only. Longitudinal accelerometry in older adults with glaucoma showed different mobility correlates across levels of fear. At higher fear levels, worsening fear was associated with fewer daily steps; at lower levels, it was associated with subsequent falls but not with step changes [14]. This heterogeneity suggests that concern does not map uniformly onto movement behavior [15]. A binary concern measure may encompass different degrees and meanings of concern, while a recent fall may vary in severity and recovery. The confidence intervals warrant caution when comparing concern-only with fall-only transitions.

Activity volume and fragmentation should also be interpreted as related but distinct dimensions. Wanigatunga et al. [13] showed that fragmentation, defined through active-to-sedentary transitions, can carry prognostic information that differs from total daily activity, underscoring that quantity and patterning are not interchangeable. In our analysis, the clearer signal was a reduction in the amount of movement; fragmentation estimates were too imprecise for firm conclusions about changes in activity patterning. The fragmentation finding is therefore better regarded as unresolved than as evidence of no change.

The restriction analysis did not identify a clear objective activity difference between restricted and unrestricted concern-only transitions. A recent systematic review found consistent associations between frailty and concern about falling, with prospective evidence also linking frailty to concern-related activity restriction [16]. This finding should not be read as evidence that concern-related activity restriction is unimportant. The restricted subgroup was small, and our analysis addressed device-measured activity change rather than disability or the development of restriction itself. More precise studies are needed to determine whether self-reported restriction identifies a distinct objective activity profile.

The design has several strengths. Repeated NHATS interviews and wrist accelerometry allowed transition states and device-measured outcomes to be aligned across three annual intervals in a person-interval design rather than inferred from a single cross-sectional assessment. Restricting each interval to a preceding-wave state of neither recent fall nor concern improved the interpretation of stable and newly observed states. Models accounted for the preceding value of each activity outcome, the complex survey design, and repeated participant contributions, and primary inference used global tests with multiplicity control. The co-occurring activity-volume estimate remained negative across alternative valid-day thresholds, change-score modeling, interval exclusions, clustering choices, and expanded sociodemographic adjustment. These features support the stability of the observed association without implying causality.

Several limitations temper interpretation. First, residual confounding and reverse causation remain possible. Prospective cohort data have linked lower objectively measured moderate-to-vigorous activity to higher subsequent fall risk, illustrating that the temporal relationship between activity and falls can also operate in the opposite direction [17]. Second, annual interviews identify a recently reported state but not its exact onset, duration, or within-month sequence, so the analysis cannot determine how rapidly activity changed after a fall or the emergence of concern. Falls and concern were self-reported, and the available measures do not capture fall severity, injury, persistence of concern, or the full range of avoidance behavior. Third, the fall-only and especially co-occurring groups were small, limiting precision and precluding detailed subgroup analyses; the restriction subgroup was smaller still. Race/ethnicity and household income were not included in the primary model because of sparse support and missingness, although expanded-adjustment sensitivity analyses did not materially alter the co-occurring activity-volume estimate. Finally, generalizability is limited by the NHATS accelerometry sample and the analytic eligibility criteria used here.

## Conclusions

Among older adults who were recently free of both falls and concern about falling, co-occurrence of the two states was associated with the clearest subsequent reduction in objectively measured activity volume. The data did not establish a clear difference between concern-only and fall-only transitions; evidence for corresponding fragmentation changes and a distinct restriction-related activity profile remained inconclusive. Keeping fall experience and concern separate, while retaining their co-occurrence as a distinct state, may help characterize subsequent free-living activity; confirmation in larger transition samples with finer temporal measurement is warranted.

## Supplementary Material

Supplementary Files

This is a list of supplementary files associated with this preprint. Click to download.

- AdditionalFile.docx
- STROBECohort.docx

## Figures and Tables

**Table 1:** Analytic sample and weighted descriptive changes by transition state

Transition state	Person-intervals	Unique participants	Activity volume change, log units	Activity fragmentation change, pp	Active minutes/day change
Stable neither	670	331	−0.03	0.61	−8.8
Concern only	140	135	−0.05	1.45	−16.42
Fall only	36	36	−0.08	1.96	−21.09
Both	27	27	−0.17	2.75	−50.93

Notes: Values are survey-weighted descriptive changes from baseline to follow-up and are not adjusted model estimates. Unique participants are counted within each transition state; because participants could contribute more than one interval and could appear in different transition states across intervals, these row-specific counts are not mutually exclusive and should not be summed. Activity fragmentation is the active-to-sedentary transition probability; pp = percentage points.

**Table 2:** Adjusted associations between transition states and subsequent objective physical activity outcomes

Outcome	Concern only vs stable neither	Fall only vs stable neither	Both vs stable neither	Global P	Holm-adjusted P
Activity volume, *%* difference	−3.18 (−7.47 to 1.32)	−6.95 (−14.16 to 0.86)	−12.41 (−20.80 to −3.14)	0.011	0.021
Activity fragmentation, pp	1.04 (−0.21 to 2.29)	1.49 (−0.85 to 3.83)	2.09 (−0.27 to 4.45)	0.080	0.080
Active minutes/day	−11.58 (−25.43 to 2.26)	−21.55 (−45.67 to 2.57)	−41.13 (−71.46 to −10.79)	0.008	–

Notes: Estimates are adjusted differences with 95% confidence intervals. Models adjust for the baseline value of the corresponding outcome, age, sex, multimorbidity count, cognition, PHQ-2, SPPB, prior-year fall history, and interval. Holm adjustment applies only to the two prespecified primary global tests (activity volume and activity fragmentation); active minutes/day is secondary. pp = percentage points.

## Data Availability

The NHATS Round 11–14 public-use datasets analyzed in this study are available to registered users through the National Health and Aging Trends Study repository, subject to the NHATS Conditions of Use. The datasets are cited in the manuscript with their persistent identifiers. No new participant-level dataset was generated for public release in this secondary analysis. The study protocol and statistical analysis plan were preregistered on the Open Science Framework (DOI: 10.17605/OSF.IO/65GF7).

## Abbreviations

ASTP: active-to-sedentary transition probability
CI: confidence interval
NHATS: National Health and Aging Trends Study
OSF: Open Science Framework
PHQ-2: two-item Patient Health Questionnaire
SPPB: Short Physical Performance Battery
